# Long-Term Toxicity of ZnO Nanoparticles on *Scenedesmus rubescens* Cultivated in Semi-Batch Mode

**DOI:** 10.3390/nano10112262

**Published:** 2020-11-16

**Authors:** Andriana F. Aravantinou, Fytoula Andreou, Ioannis D. Manariotis

**Affiliations:** Environmental Engineering Laboratory, Civil Engineering Department, University of Patras, 26504 Patras, Greece; andriana.aravantinou@gmail.com (A.F.A.); fytandreou@gmail.com (F.A.)

**Keywords:** freshwater microalgae, *Scenedesmus rubescens*, long-term toxicity, nanoparticles, zinc oxide

## Abstract

The scope of this study was to investigate the toxic effects of zinc oxide (ZnO) nanoparticles (NPs) on freshwater microalgae, in long-term semi-batch feeding mode at two different hydraulic retention times (HRTs) (20 and 40 days). A freshwater microalgae, *Scenedesmus rubescens,* was employed and exposed to a semi-continuous supply of ZnO NPs at a low concentration of 0.081 mg/L for a period of 28 d. Experiments were conducted under controlled environmental conditions. Τhe impact of ZnO NPs on *S. rubescens*, which was assessed in terms of nutrient removal, biomass growth, and algal lipid content. Semi-batch mode cultures showed that low ZnO NP concentrations at an HRT of 40 d did not have any negative effect on microalgae growth after the fourth day of culture. In contrast, algal growth was inhibited up to 17.5% at an HRT of 20 d in the presence of ZnO NPs. This might be attributed to the higher flow rate applied and ZnO NPs load. A positive correlation between nutrient removal and microalgae growth was observed. The algal lipid content was, in most cases, higher in the presence of ZnO NPs at both HRTs, indicating that even low ZnO NPs concentration cause stress resulting in higher lipid content.

## 1. Introduction

Engineered nanomaterials (NMs) are a new technology that have found wide applications in daily life during the last decade. Due to their size and antimicrobial properties, nanomaterials find applications in cosmetics, drug delivery systems, therapeutics, food packaging, biosensors, and others [1,2]. The increasing use and production of nanoparticles (NPs) has caused great concern in regulation agencies for their possible release into the environment and effect on human health and environment [2,3,4].

The extended use of NPs in numerous products results in their release into the natural environment and engineered systems. One of the systems which are affected by the increasing use of NPs are wastewater treatment plants (WWTPs). The supply of NPs in a WWTP could be continuous and cumulative. A continuous supply of NPs in a system may have a major impact on its living organisms (fungi, algae, bacteria, and protozoa) and eventually in the system operation performance. Although WWTPs are the main recipients of NPs and many studies have been performed on the effects of NPs on various organisms, the adverse effects of NPs on bacterial populations in activated sludge need further investigation [5]. Sorption of pollutants on activated sludge is among the main processes for toxic substances removal in wastewater, such as metals, synthetic organic chemicals, suspended solids, and pathogens [6], including NPs. Chauque et al. [5] investigated zinc oxide (ZnO) engineered-NPs disposal using a simulated WWTP and concluded that engineered NPs were aggregated on and attached to the biomass, and consequently, engineered NPs were removed from wastewater by sorption processes [5]. However, the behavior of NPs and especially the behavior of metal oxide NPs should be taken into consideration, since the sorption process may be ineffective due to the inhibition of bacteria by toxic substances, such as heavy metals. Large amounts of nanoparticles, mostly TiO_2_ and ZnO, are discharged into wastewater treatment plants [7]. NPs that are tightly fixed in a solid matrix are more difficult to detach than NPs that are present as free particles or agglomerates in liquid streams [8]. Among the predominant NPs, ZnO NPs rank among the top three metal NPs used in consumer products, and estimated global production ranges from 550 to 33,400 tons/year [3,8].

The toxicity of NPs depends on their physicochemical and morphological characteristics (e.g., particle size and oxidation state) [9,10], the environmental conditions (pH, temperature, and conductivity), and the microorganisms’ species [10,11]. Tsuzuki [12] reported that even the production method of NPs leads to different properties. It should be mentioned that certain properties of NPs, which might be critical to specific applications, may largely vary depending on the production techniques employed [12]. The effect of NPs on a living organism also depends on the behavior of the NPs in the living environment of organisms [3], the concentration of NPs and the exposure time [13]. The exposure time of microorganisms on NPs is a factor that has not been extensively investigated, despite the fact that NPs in nature will be present for a long period and the organisms will be exposed for a long time. Especially, metal NPs need further investigation in relation to other NPs as their toxic effect is due to their dissolution in the environment and exposure of organisms to heavy metals [3,14].

The effects of NPs on microalgae are considered an important environmental concern since they are primary producers, and undoubtedly are at the bottom of the food chain [15], as well as being the main organisms in natural wastewater treatment systems. Furthermore, algae are considered one of the most promising products for the production of biofuels, which is why many studies have focused on this area [16,17]. *Scenedesmus rubescens* is a common microalgal species which is often found in municipal wastewater and is studied for its high lipid content [16,17,18]. *Scenedesmus* sp. are often used as model microalgae for toxicity tests [10]. Various metal NPs on *Scenedesmus* sp. have been investigated including Al_2_O_3_, TiO_2_, SiC, ZnO, and CeO_2_ [2,10,11,13]. Short-term toxicity tests have shown that even at low concentrations, metal NPs have a negative effect on algal photosynthetic metabolism [10], while at high metal NPs concentrations a lethal damage of the cells may occur. On the other hand, a controlled presence of metal NPs in a *Scenedesmus* sp. culture may have a positive effect on algal growth and lipid content [10,13]. The lipid content of microalgae is an important factor for the choice of suitable algal species for biofuel production. For this reason, many studies have been focused on NPs’ effects on microalgae [8,14,19,20,21] with the latest studies focusing not on the toxic effects of NPs but in the increased lipid accumulation of microalgae due to NPs’ presence [10,22]. The effect of NP toxicity on microalgae in short term and batch mode experiments has been investigated by many researchers. However, recent studies have reported the need to study the behavior of NPs in real environmental conditions, and to relate the long-term exposure conditions as well as the continuous supply of NPs [13,21].

The aim of this study was to investigate the long-term toxic effects of zinc oxide (ZnO) NPs on freshwater microalgae, in semi-batch mode at two different hydraulic retention times (HRT). *S. rubescens* was selected as the model freshwater microalgae species, which was exposed to a semi-continuous supply of medium enriched with ZnO NPs (0.081 mg/L). Algal cells were exposed to ZnO NPs for a period of 28 days to evaluate the toxicity impact of ZnO NPs on algal metabolic reactions by determining algal growth rate, nutrient removal, and lipid production. To our knowledge no prior studies have been conducted on the exposure of microalgae to ZnO NPs for a long-term period under a semi-continuous supply of NPs. 

## 2. Materials and Methods 

### 2.1. Nanoparticles 

ZnO NPs were obtained from Sigma-Aldrich, St. Louis, MO, USA (catalogue number 544906). The particle size of ZnO NPs was smaller than 100 nm, in the range of 50 to 70 nm, and the specific surface area was from 15 to 25 m^2^/g, as reported by the manufacturer. A stock suspension of 810 mg ZnO NPs/L in deionized water was prepared and used in further experiments. The NPs suspension was dispersed before each experiment using an ultrasonic bath (Transsonic TI-H-5, Elma Hans Schmidbauer GmbH & Co., KG, Singen, Germany).

### 2.2. Microalgae

*Scenedesmus rubescens* SAG 5.95 was obtained from the *Sammlung von Algenkulturen der Universität Göttingen* (Culture Collection Algae at Göttingen University) bank (SAG). *S. rubescens* is a freshwater species and was selected due to its presence in municipal wastewater and its potential use for biofuel production because of its high lipid content. A microalgae stock cultured in ⅓N BG-11 (BlueGreen-11 enriched with one-third times the nitrates concentration: Na_2_CO_3_ (20 mg/L), NaNO_3_ (500 mg/L), Na_2_Mg Ethylenediaminetetraacetic acid (EDTA) (1 mg/L), ferric ammonium citrate (6 mg/L), citric acid∙1H_2_O (6 mg/L), CaCl_2_∙2H_2_O (36 mg/L), MgSO_4_∙7H_2_O (75 mg/L), K_2_HPO_4_ (30.5 mg/L), H_3_BO_3_ (2.86 mg/L), MnCl_2_∙4H_2_O (1.81 mg/L), ZnSO_4_∙7H_2_O (0.222 mg/L), CuSO_4_∙5H_2_O (0.079 mg/L), CoCl_2_∙6H_2_O (0.050mg/L), NaMoO_4_∙2H_2_O (0.391mg/L). The flasks were incubated under controlled environmental conditions (temperature: 21 ± 2 °C, photosynthetic radiation intensity: 150 μmol/m^2^s, and air supply: 3 L/min filtered by a 0.22 μm syringe filter) in a walk-in incubator room. Algal precultures were derived from cultures, which were in the exponential growth phase.

### 2.3. Microalgae Cultures and ZnO NPs Exposure

The toxicity experiments were performed in 2.8-L Erlenmeyer flasks using an initial cell concentration of 10^4^ cells/mL [23]. During the exponential growth phase of microalgal an appropriate volume was withdrawn and transferred to the flask, which contained sterile medium. The working volume of the cultures was 2 L. *S. rubescens* was exposed to ZnO NPs at a concentration of 0.081mg/L ZnO NPs and for HRT 20 and 40 d. Specifically, a volume of 100 or 200 mL of liquid was removed every 2 d from the flask and the same volume of enriched medium with 0.081 mg/L ZnO NPs was added for HRT 40 and 20 d, respectively. The HRT represents the time required for the full replacement of the culture volume by the substrate fed. Finally, in both cases, there was a control flask with medium without NPs. The concentration of 0.081 mg/L ZnO NPs is considered low, and has been previously investigated in short term toxicity tests [13,24].

The duration of the experiments was 28 d and the flasks were kept statically. The flasks were shaken vigorously for 30 s daily and before every sampling. Samples were taken at regular time intervals for the evaluation of microalgae growth rate, nutrient removal, and lipid content. The experiments were conducted in a walk-in incubator room at a temperature of 20 ± 2 °C. The photosynthetic radiation intensity was 100 μmol m^−2^s^−1^. Filtered air, at a flow rate of 3 L/min, was continuously supplied by an air pump (air pump, HP-400, Sunsun, Zhejiang, China). The large number of measurements and for the long period of 28 days, compared to a short-term experiment of 96 h, made difficult to conduct experiments in duplicate. From previous studies, we have seen that biomass concentration (TSS, cell numbers, chl-a), nutrients removal, and soluble Zn in short- and long-term exposure of microalgae species on ZnO NPs exhibited quite similar values. In addition, the frequent sampling during the long-term exposure and the large culture volume works beneficially for the minimization of relative errors.

### 2.4. Analytical Methods

The response of microalgae to ZnO NPs exposure was evaluated by systematic determination of algal biomass, lipid productivity, and nutrient removal, pH, turbidity and temperature. An appropriate volume of samples was withdrawn every two days for the determination of nitrates, phosphorus, chlorophyll a (chl-a), total suspended solids (TSS) and lipids. Total suspended solids and the volatile suspended solids (VSS) were determined by a gravimetric method according to standard methods [25]. Specifically, a measured volume of sample was filtered through a pre-weighed glass fiber filter. The filter was dried for at least one hour at 103 °C in an oven and the TSS were determined. Thereafter, the filter was ignited at 550 °C for 15 min in a muffle furnace to determine VSS. Due to the low ZnO NPs concentration (0.081 mg/L) used, no care was taken to separate algal cells from NPs. 

Cell number was counted in triplicate using a Neubauer hemocytometer. Ion chromatography (DX-500, Dionex Coorporation, Sunnyvale, CA, USA) was employed for the determination of nitrates, nitrites, phosphates, and sulfates. To determine the chlorophyll-a and carotenoids, a volume of the culture was filtered through a glass fiber filter, and then chlorophyll-a was extracted using 10 mL of 90% acetone, and kept at 4 °C in dark for at least for 2 h. Then the sample was centrifuged for 10 min at 10,000 rpm and 4 °C, and 3 mL of the supernatant was transferred in a cuvette and measured by spectrophotometer at 750, 665, 664 and 480 nm according to Rice et al. [25].

The modified Bligh and Dyer method [26] was employed for total lipid quantification. Briefly, approximately 100 mg of dried algal biomass (90 °C overnight) was homogenized in a mortar and extracted three times with a chloroform: methanol (2:1) mixture. The mixture was filtered through a filter paper and the extracted lipids were quantitatively transferred to a tared Erlenmeyer flask. The procedure was repeated three times. The flask was placed in an oven at 90 °C until all reagents were removed. The flask was allowed to cool to an ambient temperature in a desiccator and then was weighed. The weight difference corresponds to intracellular lipids. 

The zinc concentration of the samples was determined using atomic absorption spectroscopy (Thermo scientific, AAS-ICE 3500, Cambridge, UK) [25]. Due to the varied concentrations of Zn in the solutions, the assay was performed using air-acetylene flame or graphite furnace. Total zinc was determined according to Rice et al. [25] method after the digestion of the samples with nitric acid (65%). The soluble zinc in the samples was determined after filtering the samples with a 0.2 μm pore syringe filter. The samples were acidified with HNO_3_ (65%) to pH < 2 until measurement. 

### 2.5. Microscopy Monitoring

The interaction of ZnO NPS with the algal surface was observed by a scanning electron microscope (SEM/ microscope JEOL 6300, JEOL Ltd., Akishima, Japan) and an optical microscope (model DMLB, Leica Microsystems GmbH, Wetzlar, Germany). A volume of 0.1 to 0.2 mL of algal culture was transferred on glass slides (4.5 × 2.5 cm LxW), dehydrated in an oven and glued to SEM stubs with colloidal silver and sputter-coated with gold–palladium using a gold ion sputter coater (JFC1100 Fine Coat, JEOL Ltd., Akishima, Japan). The samples were examined with a SEM at 20 kV. The observation of algal cells by the optical microscope was conducted with untreated and treated samples with Lugol’s iodine solution. Specifically, a volume of 1 mL of algal suspension was fixed with a drop of Lugol’s iodine solution in order to immobilize the cells and make easier the microscopic observation. 

### 2.6. Data Analysis

The specific growth rate (*μ*) was determined using the following equation [23]:

ln*C_t_* = *μt* + ln*C_0_* (1)

where *C_t_* is the number of cells at time *t* (days), and *C_0_* the initial number of cells at time *t_0_*. The slope of the plot of ln*C_t_* versus *t* was used to estimate the specific growth rate (*μ*). 

The growth inhibition rate (I%) of microalgae was calculated according to the OECD 201 guideline [23]:

Inhibition (%) = [(*μ_control_* − *μ_toxicity_*)/*μ_control_*] × 100 (2)

where *μ_control_* is the mean value of average specific growth rate (*μ*) in the control, and *μ_toxicity_* is the average specific growth rate for the treatment replicate. It should be noted that the culture flasks were sampled twice and measured in order to determine the cells number, which was used to calculate specific growth rate and growth inhibition rate values. 

## 3. Results

### 3.1. HRT and ZnO NPs Effect on Algal Growth 

**Figure 1:**
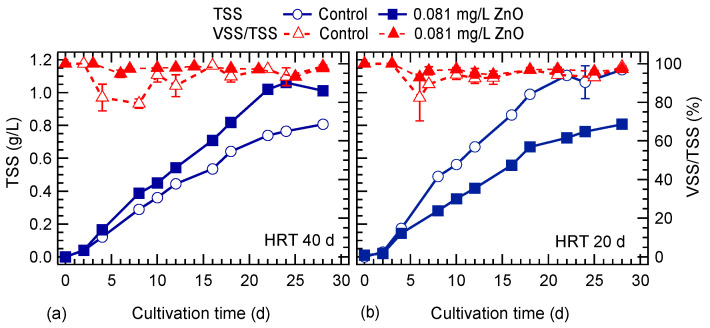
Effect of ZnO NPs on *S. rubescens* biomass concentration in terms of total suspended solids (TSS) and volatile suspended solids (VSS) cultured in ⅓N BG-11 medium at hydraulic retention time (HRT) of: (**a**) 40 d and (**b**) 20 d.

The algal biomass concentration in terms of TSS and VSS is shown in Figure 1. At an HRT of 40 d, the TSS concentration was higher in the culture exposed to ZnO NPs compared to the control culture. The opposite was observed with the lower HRT of 20 d. It seems that at the higher HRT, ZnO NPs had a positive effect on algal growth, while at the lower HRT, the presence of ZnO NPs resulted in lower TSS concentration, indicating their impact at higher flow rates on algal growth. The average VSS content of the biomass was about 94% and 97% for the control and ZnO exposed cultures, for the two HTRs examined. As it is seen, the algal TSS content was higher in the culture exposed to ZnO NPs than VSS, at both HRTs.

**Figure 2:**
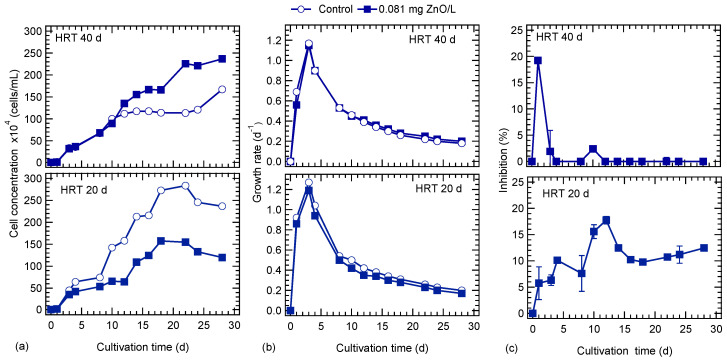
Effect of ZnO NPs on *S. rubescens* cultivated in ⅓N BG-11 medium at two hydraulic retention times (HRT): (**a**) cell concentration, (**b**) growth rate and (**c**) inhibition growth rate (%*I*).

Figure 2a shows the cell concentration of cultures for each HRT studied. At an HRT of 40 d, the cell concentration of cultures in the presence and absence of ZnO NPs was similar for the first 10 days of cultivation, and thereafter, a higher number of cells was observed for the culture exposed to 0.081 mg/L ZnO NPs. This behavior was similar to TSS concentration profile (Figure 1). In the 0.081 mg/L ZnO NPs culture, a first plateau was observed during the period from 12 to 18 days and a second from 22 to 28 days. In the control culture a stationary phase was observed after the 12th day and started to increase only at the end of the operation period. The maximum concentration of cells was 1.67 × 10^6^ and 2.37 × 10^6^ cells/mL at the end of cultivation period in the control and ZnO NPs exposed cultures, respectively. At the lower HRT of 20 d, even from the first day of operation, a higher cell concentration was observed in the control culture. The maximum cell concentration was 2.83 × 10^6^ and 1.58 × 10^6^ cells/mL after 22 and 18 days in the control and ZnO NPs exposed cultures, respectively. Thereafter, the cell concentration started to decline in both cultures. The HRT had a significant effect on the growth of *S. rubescens* during semi-batch culture conditions; at a low HRT, the maximum cell concentration occurs earlier compared to the higher HRT. This is attributed to the nutrient and micronutrient supply to the cultures. Specifically, with low HRT (20 d) the amount of nutrients that was fed to the cultures was higher than in the HRT of 40 d. That means that a higher amount of phosphates was fed to cultures at HRT of 20 d, which led to the early high peak of the algal growth. This resulted in a faster growth rate of algae at low HRT, as it is shown in Figure 2b. The maximum growth rate occurred in all cultures on the third day of cultivation and was higher in cultures at an HRT of 20 d compared to the 40-d HRT cultures. Specifically, in the control culture the growth rate of microalgae was 1.17 and 1.27 d^−1^ for HRT 40 and 20 d, respectively (Figure 2b). 

Figure 2c illustrates the growth inhibition of *S. rubescens* exposed to ZnO NPs. In the case of 40-d HRT, the maximum inhibition was about 20% in the culture exposed to ZnO NPs during the first days and after the 4th day inhibition was practically zero. The latter implies that the 0.081 mg/L ZnO concentration was low to cause any long-term toxicity on algal growth, and after a short period *S. rubescens* acclimated to the presence of nanoparticles. On the other hand, at the lower HRT (20 d) culture, ZnO NPs had a more severe impact on algal growth and the inhibition reached up to 18% on the 12th day of operation, and thereafter decreased. Toward the end of operation inhibition was around 12%. 

**Figure 3:**
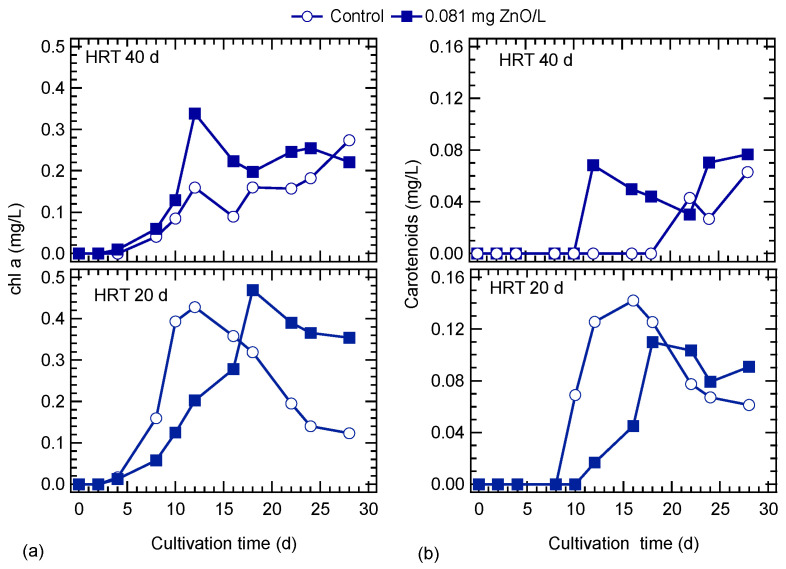
Effect of ZnO NPs on (**a**) chl-a and (**b**) carotenoids content of *S. rubescens* cultivated in ⅓N BG-11 medium at two hydraulic retention times (HRT).

After a lag period of four days, chl-a started to increase in cultures at both HRTs. At an HRT of 40 d, the chl-a was higher in the ZnO exposed culture, while at the end of the operation period, chl-a was higher in the control. The maximum chl-a concentration was observed in the cultures at an HRT of 20 d and was 0.43 and 0.47 mg/L on 12th and 18th day, in the control and ZnO exposed cultures, respectively (Figure 3a). At an HRT of 20 d, after the peak, chl-a started to decline and the higher drop was in the control culture. Carotenoids started to increase around the 10th day for both HRTs in the ZnO exposed cultures (Figure 3b). The appearance of carotenoids in the control cultures seems to be affected by the HRT, and their concentration started to increase after 22 and 10 d at HRTs of 40 and 20 d, respectively. Generally, higher carotenoid concentrations were observed for cultures operated at the lower HRT. 

### 3.2. ZnO NPs Effects on Nutrients Uptake

pH values ranged from 8.2 to 11.2 in all cultures and generally, a similar trend was observed regardless the ZnO NPs’ presence and the HRT applied. After the fifth day, the pH increased to over 10 in all cultures, and thereafter there was a slight increase until the 18th day. From the 18th day, a drop was observed and at the end of the operation, the pH was around 9.7.

**Figure 4:**
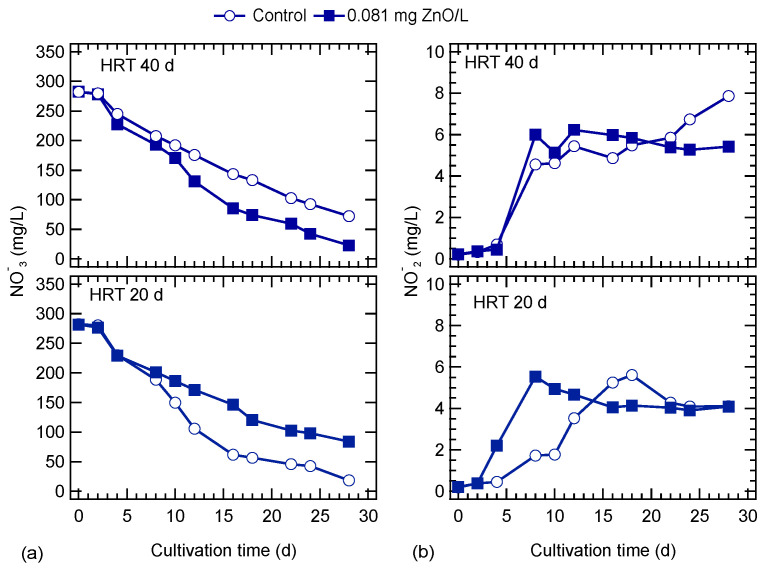
Effect of ZnO NPs on (**a**) nitrates and (**b**) nitrites concentration of *S. rubescens* cultures in ⅓N BG-11 medium at two hydraulic retention times (HRT).

**Figure 5:**
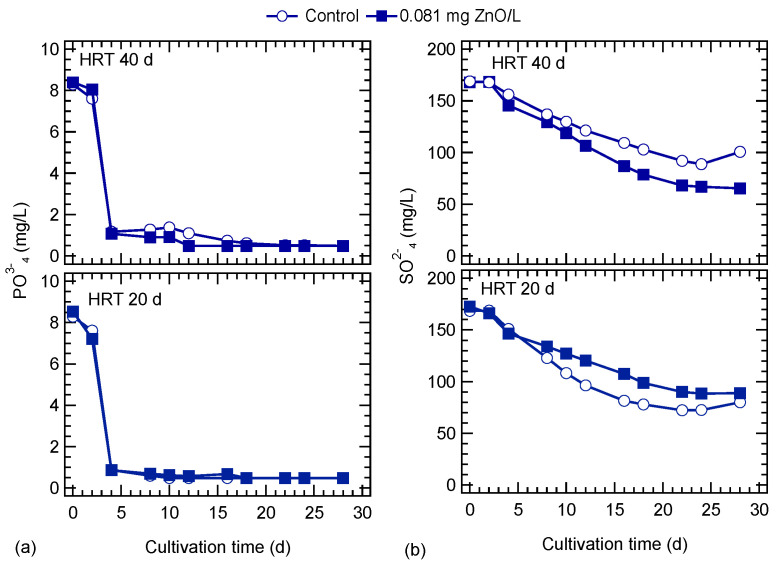
Effect of ZnO NPs on (**a**) phosphates and (**b**) sulfates concentration of *S. rubescens* cultures in ⅓N BG-11 medium at two hydraulic retention times (HRT).

The variation of nitrates and nitrite concentrations during the exposure to ZnO NPs is shown in Figure 4. Nitrates gradually decreased during cultivation. At a 40-d HRT, the decrease in nitrates was higher in the ZnO-exposed culture, compared to the control. The opposite behavior was observed for the cultures at an HRT of 20 d. Nitrite concentration gradually increased for the ZnO-exposed and control cultures at the HRT of 40 d up to the eighth day, and thereafter, a plateau was observed for the ZnO exposed culture. In the culture with HRT of 20 d, nitrite concentration was gradually increased up to the 8th and 18th day for the ZnO-exposed and control cultures, respectively. At the end of the operation, nitrite concentrations reached the same level in both cultures. Phosphates were rapidly consumed; even from the fourth day of cultivation, their concentration was below 1 mg/L (Figure 5a). Sulfates were consumed in a similar way as nitrates, and the culture exposed to ZnO NPs at the lower HRT showed a slightly higher consumption (Figure 5b). 

### 3.3. ZnO NPs Effect on Lipid Content

**Figure 6:**
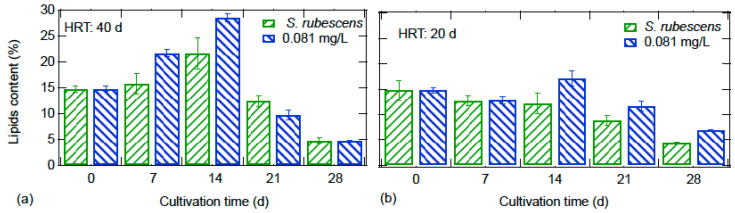
Effect of ZnO NPs on *S. rubescens* biomass lipid content at hydraulic retention times (HRT) of: (**a**) 40 d and (**b**) 20 d.

The lipid content of algal biomass is presented in Figure 6. At the HRT of 40 d, a maximum lipid content of 28% and 22% was observed on the 14th day ZnO exposed and control cultures, respectively. Thereafter, the lipid content gradually decreased. At an HRT of 20 d, a smooth decrease in lipid content is observed after the 14th day of operation. Generally, the cultivation at the lower HRT resulted in lower lipid content of algal biomass.

### 3.4. ZnO NPs Behavior 

**Figure 7:**
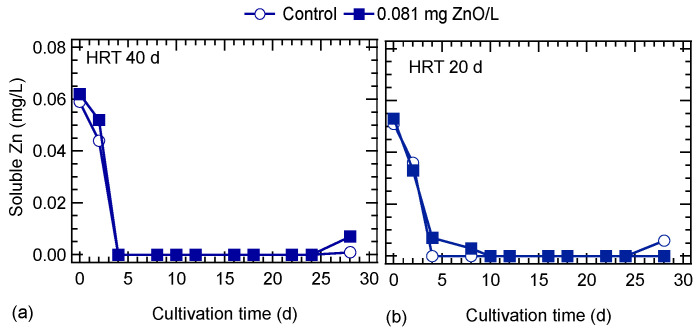
Soluble Zn concentration of *S. rubescens* cultures at hydraulic retention times (HRT) of: (**a**) 40 d and (**b**) 20 d.

The concentration of soluble Zn in cultures is shown in Figure 7. The Zn concentration in cultures at the beginning of the experiment was around 0.06 and 0.05 mg/L at an HRT of 40 and 20 d, respectively. It should be noted that the Zn content of the in ⅓N BG-11 medium was 0.05 mg/L. It seems that the 0.081 mg/L ZnO NPs added, which are equivalent to 0.065 mg Zn/L, were not in soluble form and formed either agglomerates or were adsorbed onto algal cells. After the fourth day of operation the soluble Zn was almost zero in all cultures (Figure 7). This means that the soluble Zn was consumed or adsorbed by algae cells immediately, which was confirmed by the total Zn concentration. In cultures without ZnO NPs (control) the total Zn concentration was measured with the same frequency as soluble Zn, and was around 0.058 mg/L at both HRTs. Similarly, in cultures exposed to ZnO NPs the total Zn was 0.116 ± 0.014 mg/L and 0.109 ± 0.024 mg/L at HRT of 40 and 20 d, respectively. After the 20th day of operation the total Zn concentration started to increase reaching values on the 28th day of 0.15 and 0.17 mg/L for the cultures exposed to ZnO NPs at HRT of 40 and 20 d, respectively. This increase may be attributed to the lower photosynthetic activity and consequently lower zinc assimilation by microalgae. 

**Figure 8:**
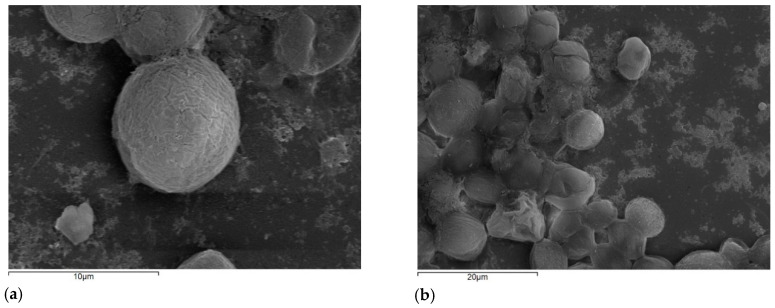
Representative SEM images of *S. rubescens*, after exposure of 0.081 mg/L ZnO NPs for 14 days at a magnification of (**a**) 5000× and (**b**) 2000×.

SEM images analyses (Figure 8) did not confirm the form of any ZnO NPs agglomerates or deposition on the algal surface, probably due to the low concentration of ZnO NPs which was applied and their dissolution in the medium. The size distribution of ZnO NPs at a concentration of 0.081 mg/L in ⅓N BG-11 for a period of 4 days showed two distinct populations, one with a mean peak of several nanometers (around 70 nm), and the presence of larger aggregates with a mean peak of several hundred nanometers (>200 nm). The size of larger aggregates was decreased with time from 850 nm (day 0) to 270 nm (day 4) [11]. The soluble Zn concentration of both cultures (with and without ZnO NPs) operated at a 40-d HRT was zero on the fourth day of operation. At an HRT of 20 d, the soluble Zn concentration was zero after the fourth and eighth day for the control and ZnO-exposed cultures, respectively. 

## 4. Discussion

### 4.1. HRT and ZnO NPs Effect on Algal Growth

The increasing use of NPs in daily products leads to their release in WWTPs and natural systems. Although the environmental concentrations of NMs are still unknown, there is evidence of the presence of NPs in WWTPs [27], from which some of them escape into water bodies [28]. NPs may remain in the environment for a long period and can be potentially toxic to aquatic life [28]. According to previous studies, the impact of ZnO NPs on microalgae growth differs between short- and long-term monitoring [13]. Long-term exposure of algae to NPs simulates better the exposure of microalgae to NPs in natural environment.

The impact of nanomaterials on wastewater treatment processes is largely unknown [28]. Even though many studies have investigated the impact of NPs’ presence in activated sludge systems [5,28], there is little information on their impact on natural treatment systems. Waste stabilization ponds are common natural systems, and their operation is based on bacteria and algae symbiosis. The HRT in these systems is an important factor for the performance of the process and supply of nutrients.

The present study revealed that HRT has a considerable effect on *S. rubescens* growth during semi-batch culture conditions. Specifically, at a high HRT (low flow rate), ZnO NPs had a positive effect on algal growth, while the low HRT resulted in lower biomass concentration. The latter implies that the high substrate feeding rate (low HRT) is crucial for the toxic effect of ZnO NPs. It should be mentioned that ZnO NPs at low concentrations may have been less agglomerated and induced higher growth inhibition [13]. The results of the biomass concentration point to the suggestion that *S. rubescens* had higher demand for zinc than the ⅓N BG-11 medium could offer, and in the presence of ZnO NPs, the algal growth was enhanced. Zinc is an essential micronutrient for algal growth, and is required in many biological processes since it is present in nearly 300 enzymes, which perform many special metabolic functions [29,30,31]. Starodub and co-workers [32], also reported that Zn at higher concentrations may stimulate algal metabolic activity. In addition, improved growth rates at intermediate and higher levels of Zn, reported in the literature, are probably attributed to the function of Zn as an essential nutrient [32]. Furthermore, Shehata and Badr [33] investigated *Scenedesmus sp*. growth at different Zn concentrations and showed that there was a slight increase of algal growth at Zn concentrations up to 1 mg/L. In previous research [11], *S. rubescens* was exposed to 8.1 mg ZnO NPs/L. The zinc content of algal cultures was lower (87%) than that of the control (without microalgae) indicating the uptake of zinc by microalgae.

Photosynthesis is an important physiological process of algal survival in the aquatic environment [34]. Previous studies [13,34] have mentioned that the adsorption of NPs on the algal surface may result in a shading effect that affects algal photosynthesis. The shading caused by NPs restricts the absorption of light by the cell, which results in the inhibition of the photosynthesis process [34]. However, scanning electron microscopy (SEM) images of this study did not confirm the NPs’ deposition on the algal surface, probably due to the low concentration of ZnO NPs which was applied and their uptake by algae. Nevertheless, the presence of NPs may have an effect on photosynthetic activities because of reactive oxygen species (ROS) production. Wang et al. [34] reported that in the presence of graphene oxide NPs the photosynthetic rate of algae was changed, resulting in a metabolic disturbance. Similar results were observed in the present study in the case of low HRT (20 d) where the algae were affected by the presence of ZnO NPs and the chl-a peak concentration was on the 18th day of the exposure time, whereas in the control, it was observed six days earlier.

The presence of low ZnO NP concentration may affect algae growth during long-term exposure. The concentration of NPs in the aquatic systems is still low. However, NPs tend to accumulate in high trophic level organisms and can cause significant toxic effects by the stepwise delivery or enrichment of the food chain. The transmission of NPs in the different levels of the food chain of the aquatic ecosystem, as well as the impact of environmental factors on this transmission, is still limited [34].

### 4.2. ZnO NPs Effects on Algal Nutrients Uptake and Lipid Accumulation

pH variation and increase depends on algal biomass production. pH changes were expected due to the metabolic activities of algal cells, which cause an increase in pH [19]. The pH of algae cultures increases with photosynthesis, as algae continue to consume CO_2_ faster than it can be transferred via the air-water interface. As the diffusion of CO_2_ from the air is quite low, and the simultaneous consumption of CO_2_ from algae increases, the concentration of hydroxyl ions in the water column causes the increase oin pH above 10 [35].

It is important to assess the effects of metal nanoparticles on nutrient removal in biological wastewater treatment processes [27]. During the exposure of *S. rubescens* to ZnO NPs, it was observed that nutrient removal was analogous to biomass concentration, and nutrient uptake was not affected by the presence of NPs. This was confirmed by nutrient concentration normalized to biomass (data not shown). Specifically, nitrates in the control cultures were faster consumed at HRT of 20 d compared to HRT of 40 d, and this was supported by the early appearance of nitrites on the fourth day of cultivation. The faster removal of nitrates in the low HRT (20 d) culture was as a result of the higher supply of phosphates in the cultures. BG-11 is a low phosphate-content medium that makes it the limiting factor of algal growth. Similar results were presented by Ernst et al. [36], who cultivated two strains (BO 8807, and SAG 3.81) of *Synechococcus* in BG-11 medium and observed that the initial phosphate concentration used in BG-11 inhibited the growth of both strains.

On the contrary, the presence of ZnO NPs seems to be beneficial on the lipid content of *S. rubescens*. Specifically, the exposure of algae on ZnO NPs enhanced lipid accumulation despite the high concentration of nitrates in the cultures. Many studies [21,22] nowadays have focused on enhancing the lipid accumulation of microalgae by adding NPs, in an effort to reduce the cost of biofuel production. Ren and co-workers [22] investigated the boost of lipid accumulation of *Scenedesmus sp*. using TiO_2_, TiC, SiC, and g-C3N_4_ nanoparticles. SiC nanoparticles were more efficient in lipid accumulation of *Scenedesmus sp*., when it was cultivated under xenon lamp illumination [22].

### 4.3. Evaluation of Toxicity

The toxicity of NPs on algae is associated with biotic and abiotic factors, which could be grouped into three major categories; 1. Environmental conditions (pH, conductivity, temperature, light, nutrients, etc.); 2. Algal charactesristics (species, strain, morphology, membrane, size, time, surface functional groups, etc.); 3. NP type (size, particle diameter, shape, surface morphology, chemical composition, synthesis method, aggregation state, concentration and surface chemistry, time, surface functional groups, solubility and dispersion of the nanoparticles, etc.) [34]. NPs’ environmental fate and transport may be affected by many physicochemical properties (i.e., size). NPs may undergo transformations in an aquatic medium, which may affect their physical or chemical nature. For instance, mineral-based NPs can undergo chemical or biological oxidation, which results in modified functional group [37]. Particle size is also affected by the initial ZnO NP concentration as well as the solution composition and the exposure period [11].

Another important factor of NP toxicity is the exposure time. Previous studies have reported that short and long-term exposure periods may have different results of the toxic effect of NPs on algae [13,32]. Algal cells exposed to short-term pressures may exhibit temporary disturbances and long-term studies would provide adequate time for physiological adjustment and adaption [32].

The fate and behavior of NPs in the environmental condition play a key role in the toxic effects on algal cells. An important factor of ZnO NPs that determines their toxic effect on algae is their solubility and dispersion into aquatic matrix. The determination of dissolved zinc is essential in order to estimate the effects of toxicity on species due to the dissolved zinc, and the effects that arise from nanoparticle suspensions [11]. In the present study, the filtered Zn concentration was lower than estimated, which indicates that ZnO NPs in the 1/3 BG-11 medium were bound in agglomerates or were sorbed onto algal cells. SEM images during the first days did not show agglomerates of NPs or any attached NPs on the surface of the algal cells. This implies that the ZnO NPs were absorbed by algal cells. However, the toxicity of ZnO nanoparticles is not clearly attributed to the release of zinc ions nor to their small size, and for this purpose further investigation is needed to better determine the effect of ZnO NPs on algal growth.

Peng et al. [38] reported higher release of Zn^2+^ ions from spherical structures compared to rod structures of ZnO NPs. Leung et al. [39] revealed that the modification of NPs’ surface could influence the release of Zn^2+^ ions and ROS production. Many studies have reported that the major toxicological concern is that some of the manufactured nanomaterials are transported across algal cell membranes, especially into mitochondria [28]. Concequenlty, NPs may cause damage of algal cells due to the accumulation of nanoparticles in the membrane, and ROS production, which subsequently impact macromolecules, including DNA, lipoproteins, and enzymes [27]. Agarwal et al. [40] reported that ZnO NPs interact with the cell membrane and may form pits with intracellular leakage, which eventually causes cell death. ROS production can cause significant damage in the cell such as loss of fluidity through the membrane, and oxidation of unsaturated lipids [20]. The oxidative damage mechanism has been considered to be one of the major possible mechanisms for NP ecotoxicity to algae, which can cause damage to the cell wall, cell membrane, and organelles [34].

Furthermore, for very small particles (< 20 nm), internalization can be visualized only by the TEM technique. NP uptake seems to occur naturally via cell wall pores or via mechanical destructuring. The entrance of particles inside the cell may modify the gene expression of microalgae [20], which can cause permanent damages to algal cells. Simon et al. [41] reported for *Chlamydomonas reinhardtii* a decrease at the transcript level linked with photosynthesis (growth reduction) in the presence of titanium dioxide or zinc oxide NPs, and a rise in gene transcripts linked to proteasome (increase in detoxification system, proteins bend incorrectly). This study also underlines an enhancement of transcript levels encoding cell wall components in the presence of silver NPs [41].

Finally, it is worthy to note that Aravantinou and co-workers [13] examined the long-term exposure of *S. rubescens* to ZnO NPs at a concentration of 0.081 mg/L, in batch mode conditions, and reported that the inhibition of algal growth was increased during the cultivation period due to the lack of nutrients, which can cause oxidative stress on cells. In the present study, the cultures operated at an HRT of 40 d have a low supply of ZnO NPs which was closer to the batch mode conditions in Aravantinou et al.’s [13] study. The growth inhibition rate was practically zero after the 10th day of operation (Figure 2c) indicating that the lack of nutrients enhances the inhibition of ZnO NPs.

## 5. Conclusions

The results of the present study revealed that algal growth was significantly affected by the exposure time and the NPs supply in the system. Even though the concentration of ZnO NPs was low (0.081 mg/L), toxic effects on algal growth were observed at the low HRT (20 d). The inhibition of growth rate reached values up to 18% on the 12th day of operation and decreased thereafter. Moreover, this study confirmed that a lack of nutrients enhances the inhibition of ZnO NPs. The stress in the presence of ZnO NPs enhanced the lipid content of *S. rubescens* at both HRTs, despite the fact that in case of the high HRT, no negative effects on algal growth and nutrient removal were observed. The highest lipid content was 28.5 and 22.6% at an HRT of 40 days in the presence and absence of ZnO NPs, respectively. The present work revealed the need for the investigation of the NPs’ effects on microalgae in real environmental conditions of a natural wastewater treatment system. Further research should be focused on long-term NP exposure, including autochthonous wild strains that are of greater importance for the local community.
